# The impact of sensory impairments and eye diseases on cognitive function in elderly Chinese: The mediating effects of social participation

**DOI:** 10.7189/jogh.13.04068

**Published:** 2023-07-28

**Authors:** Xiuxiu Zhou, Hong Wu

**Affiliations:** 1 Department of Psychiatry, Wuhan Mental Health Center, Wuhan, Hubei province, China; 2 Department of Psychiatry, Wuhan Hospital for Psychotherapy, Wuhan, Hubei province, China; 3 School of Medicine and Health Management, Tongji Medical College, Huazhong University of Science and Technology, Wuhan, China

## Abstract

**Background:**

Sensory impairments and eye diseases increase the risk of cognitive decline, but little is known regarding their influence on cognitive function in elderly Chinese and the underlying mechanisms. We aimed to explore these influence mechanism from the social participation perspective.

**Methods:**

We selected 2876 respondents aged ≥60 from the China Health and Retirement Longitudinal Study (CHARLS) conducted in 2013, 2015, and 2018. We assessed sensory impairments and eye diseases based on self-reported responses, and evaluated its relation to social participation and cognitive function by fixed-effects regression and mediation effect analysis over a five-year period.

**Results:**

Respondents with visual impairment and cataracts had poor memory and mental status. Compared with near visual impairment, distance visual impairment was associated with a 1.7 times higher likelihood of cognitive decline (correlation coefficient (β) = -0.051; 95% confidence interval (CI) = -0.065, -0.036)). Respondents with hearing impairment had bad memory (β = -0.046; 95% CI = -0.065, -0.036), but not mental status. Social participation partially mediated the relationships of sensory impairments and cataracts with cognitive function in elderly Chinese. Individuals with sensory impairments affected by limited social participation reported a faster cognitive decline compared to those with eye disease.

**Conclusions:**

We found that sensory impairments and eye diseases were negatively associated with cognitive function. Furthermore, sensory impairments and cataracts influence cognitive function partly via social participation. Our results have important theoretical and practical implications and suggests that early interventions for sensory impairments and eye diseases may improve the cognitive function of elderly people.

With the rapid population ageing worldwide, dementia has become a global public health challenge. An estimated 46.8 million people were living with dementia in 2015 [1], a number which is expected to increase to 75 million by 2030 and to 132 million by 2050 [2]. China has the world’s largest elderly population (≥60 years old) [3], and its dementia-affected population is expected to reach 10 million by 2050 [4], increasing the burden for individuals, families, and health care systems. Considering cognitive decline is a common neurodegenerative change and an important early clinical feature of dementia [5]. Additionally, as the symptoms progressively worsen, 8%-25% of individuals with mild cognitive impairment develop dementia in the following three to six years [6]. Therefore, it is important to identify the risk factors and explore interventions for mild cognitive impairment for the early identification and prevention of dementia.

As hearing and vision degrade with ageing, impaired vision and hearing are most common among older adults [7]. It is estimated that more than 900 million people worldwide will have clinically significant hearing loss by 2050, and about 2.2 billion will be living with some form of visual impairment, including its moderate to severe manifestations and blindness [8]. Sensory impairments (including visual and hearing impairment) have been found to be associated with physical function, daily life, and mental health [9], possibly because they are chronic conditions, which is why affected individuals show lower accuracy, higher reaction times, and lower sensitivity, possibly increasing the risk of cognitive decline [10,11]. For example, a long-term prospective study using data from the Health and Retirement Study (HRS) found that individuals aged 50-98 years with sensory impairments at baseline had a 38% higher risk of cognitive decline than individuals without sensory impairments [10]. According to a cross-sectional study of 13 914 Chinese adults aged ≥45 years from the China Health and Retirement Longitudinal Study (CHARLS), vision and hearing impairment were negatively associated with cognitive function [12]. Furthermore, a systematic review and meta-analysis found that age-related hearing loss was a possible biomarker and modifiable risk factor for cognitive decline, cognitive impairment, and dementia [11]. However, a multicentre study using data from the Study of Osteoporotic Fractures found that women aged ≥69 years with visual impairment, but not hearing impairment, had an increased likelihood of cognitive decline [13]. A cohort study on 8939 participants aged 50-95 years from the USA found eye diseases, including glaucoma and cataracts, were not significantly associated with memory decline [9]. However, another study involving 1659 residents aged ≥60 found cataracts and glaucoma were positively associated with cognitive decline [14]. While prior studies have come to seemingly consistent conclusions, they have only focused on sensory impairments or eye diseases, or one dimension of cognitive function, and have used cross-sectional data, thus preventing conclusions regarding causality. Additionally, it is unclear whether sensory impairments or eye diseases affect cognitive function in old age, as studies have not explored the underlying mechanism. Furthermore, as China has the world’s largest elderly population, limited resources, and restricted primary medical and health care [3], the health conditions of the elderly Chinese with sensory impairments or eye diseases may be more serious than in western countries.

Consequently, by collecting longitudinal data from a nationally representative sample of adults aged ≥60 years in China, we explored the impact of sensory impairments and eye diseases on cognitive function by analysing specific types of sensory impairments (including near visual, distance visual, and hearing impairment), eye diseases (including cataracts and glaucoma), and two dimensions of cognitive function (memory and mental status). We focused on two research questions:

Q1: Will sensory impairments or eye diseases affect the cognitive function of Chinese older people? Are their influences different?

Q2: How do sensory impairments or eye diseases impact cognitive function? What is the role of social participation?

## Theoretical background and hypotheses development

### Sensory impairments, eye diseases and cognitive function

The prevalence of sensory impairments and eye diseases in China was reported to be much higher than in Western Europe and the USA [9]. Sensory impairments, including visual and hearing impairment, affect many aspects of an individual’s life. Recent evidence has demonstrated that they are associated with a higher risk of cognitive impairment [12]. Per the cognitive load on perception hypothesis, cognitive decline can impose a harmful cognitive load on perceptual functioning [8], meaning that individuals under high cognitive load suffer from sensory impairments due to limited cognitive resources. Moreover, the information degradation hypothesis suggests that decreased perceptual information can decrease neuronal input, potentially accelerating neurodegeneration and resulting in cognitive decline among older adults [15]. Furthermore, the common cause hypothesis indicates that a common cause results in both age-related cognitive decline and sensory impairments in the process of neural degeneration, i.e. that impairments and cognitive decline may be caused by β-amyloid pathology [8].

Additionally, several studies have shown that eye diseases are associated with cognitive impairment. A study on 1659 individuals aged ≥60 years with a mean 5.2-year-follow-up indicated that cataracts and glaucoma may lead to an increased risk of cognitive load, brain structural damage, and cognitive decline due to the loss of visual input and decreasing activation in central sensory pathways [14]. In a sample of 3038 participants aged ≥65 years from the Adult Changes in Thought study in Washington, cataracts were significantly associated with a higher risk of dementia development [15]. The could be explained by the fact that cataract-related visual impairment decreases neuronal input, potentially accelerating neurodegeneration or magnifying its effect through cortical atrophy, eventually leading to cognitive decline.

Despite increasing evidence for an increased risk of cognitive decline from sensory impairments and eye diseases, few studies investigated their causal relationship. Furthermore, most studies have been conducted in Western countries, and little evidence is available on this association among older Chinese. Since both theories and empirical evidence have generally suggested that sensory impairments or eye diseases are associated with cognitive decline, we hypothesised that:

Hypothesis 1a: Chinese older people who suffered from sensory impairments develop worse cognitive function over time.

Hypothesis 1b: Chinese older people who suffered from eye diseases develop worse cognitive function over time.

### Sensory impairments, eye diseases and social participation

Hearing and vision function are integral to quality of life, affecting mobility and communication, and thus, an individual’s social participation. In a sample of 21 241 participants from the Canadian Longitudinal Study on Aging tracking cohort [16], researchers found that visual impairment and hearing impairment were associated with reduced social function in older Canadians. In a study involving 229 participants from Malaysia aged ≥60 years, loss of hearing and vision may significantly reduce the participation of older adults in activities of daily living, such as visually intensive tasks and social activities [17]. Additionally, a longitudinal study of Americans aged 70 years and older showed individuals with sensory impairments were more likely to be depressed, struggle with activities of daily life, and had limited social participation [18]. Older people with visual impairment and hearing impairment may appear timid, hesitant, and confused, especially when confronted with a new situation [18]. These experiences may result in less social interaction and less communication with the outside world. According to the sensory deprivation hypothesis [8], visual impairment and hearing impairment may result in neuroplastic changes, depression, and social isolation, ultimately leading to a decline in social participation. Thus, we hypothesised that:

Hypothesis 2a: Sensory impairments negatively affect social participation among Chinese older people.

Hypothesis 2b: Eye diseases negatively affect social participation among Chinese older people.

### Social participation and cognitive function

With the deepening of the concept of healthy ageing, researchers are becoming attentive of the elderly population’s social participation. It is shaped by the sociocultural context in which older adults are embedded, which shapes both the extent and types of social engagement and the compatibility of different activities; furthermore. Chinese culture is family-oriented, advocating filial piety and human kindness, and mutual help is a cultural norm [19]. “The five constant relationships” refer to the five fundamental relationships in Confucian philosophy: those between ruler and subject, father and son, elder brother and younger brother, husband and wife, and two friends [20]. The five relationships constitute the basic hierarchical structure of Confucian society. Compared with the elderly worldwide, Chinese older people especially need to socialise.

According to the activity theory [21], healthy aging requires the continuation of interests and activities started in mid-adulthood and the maintenance of the number and type of social interactions, emphasising the importance of social participation. With aging and retirement, the social value of the elderly seems to decrease and their social network shrinks. Social participation compensates for this by providing social relationships and resources, and building new social networks to expand their existing ones. Moreover, the role theory [22] suggests that the sudden role loss individuals encounter when they move into older age may result in negative effects, especially when access to new roles is not concurrently available; contrastingly, role enhancement hypothesises that a collection of multiple participating roles, due to opportunities for engaging with others through social networks, may lead to better well-being and quality of life among older adults. Thus, social participation may be more necessary than ever for older people.

Prior literature indicates that social participation is a potentially in maintaining cognitive function [23]. The “use it or lose it” hypothesis suggests that participating in social activities may increase one’s cognitive activities that contribute to larger cognitive stimulation and better cognitive function [24]. The cognitive reserve hypothesis suggests that participating in social activities may increase the efficiency and plasticity of related neural networks, possibly slowing cognitive deterioration [25]. A prospective cohort study including 9008 individuals aged ≥65 years or older from the Canadian Study of Health and Aging showed participating in social activities may help accelerate cerebral blood flow, while increasing aerobic capacity and cerebral nutrient supply, thus reducing the risk of cognitive impairment [26]. Another review showed social participation may help increase interpersonal interaction and social support, and decrease stress reactivity and anxiety thoughts, resulting in better cognitive performance [27]. Therefore, social participation may be an important mechanism for maintaining cognitive function in the elderly. We hypothesised that:

Hypothesis 3: Social participation positively affects cognitive function among Chinese older people.

### Sensory impairments, eye diseases, social participation and cognitive function

With the accumulation of experience in healthy integration and disease prevention, the treatment of diseases has gradually changed from a simple biomedical model to a bio-psycho-social medical model, so as to prevent and treat diseases in a more comprehensive way. Healthy ageing is not only related to individual behaviours, but to society overall. A longitudinal study including 4267 participants aged ≥65 years in China showed engaging in leisure activities mediated the relationship between hearing difficulty and cognitive impairment, indicating social participation may be a key pathway linking hearing difficulty and cognitive impairment [28]. Another study including 8199 individuals aged ≥50 years conducted in England showed the link between hearing impairment and cognitive function was partly mediated by social isolation, which manifests improving the social networks of older adults with hearing impairment are likely to be beneficial in preventing cognitive decline [29]. Although prior studies have found that the relationship between hearing impairment and cognitive function is mediated by social participation, they are limited by focusing on hearing impairment only, and these studies have used different measurements of hearing impairment. Additionally, current studies focused on middle-aged and elderly people aged ≥50, so there is a lack of data for older people in China aged ≥60 years. Evidence suggests that older people with sensory impairments or eye diseases appear depressed and fearful, which limits their social participation [17,18]. Meanwhile, the decreased cognitive activities arising from participation in social activities may contribute to less cognitive stimulation and worse cognitive function [19]. Based on existing studies, we hypothesised that:

Hypothesis 4a: Chinese older people with sensory impairments affected by limited social participation (hypothesis 2a) would report faster cognitive decline (hypothesis 3).

Hypothesis 4b: Chinese older people with eye diseases affected by limited social participation (hypothesis 2b) would report faster cognitive decline (hypothesis 3).

**Figure 1:**
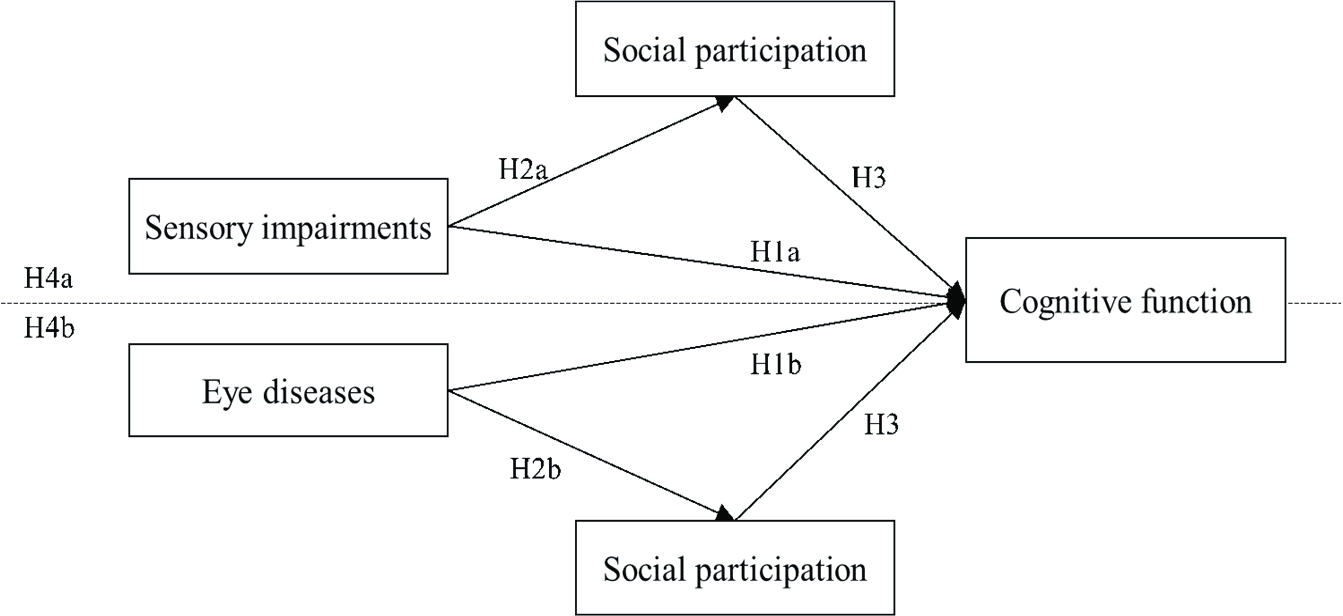
Research model and hypothesised relationships.

**Figure 1** shows the research model and hypothesised relationships.

## METHODS

### Study sample

**Figure 2:**
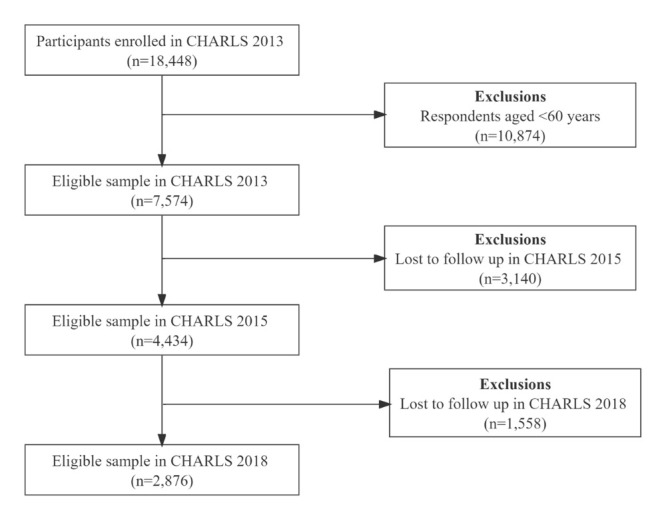
Flowchart of participant selection.

We used data from the 2013, 2015, and 2018 CHARLS, a nationally representative longitudinal survey of Chinese individuals aged ≥45 years and their spouses, which also assessed the social, economic, and health circumstances of community residents [30]. The surveys were conducted by the China Centre for Economic Research of Peking University, with a probability-proportional-to-size sampling technique covering 450 villages and 150 counties in 28 provinces. The study collected data through face-to-face interviews. We selected 2876 respondents aged ≥60 years for the fixed-effects regression and mediation effect analysis (**Figure 2**). We implemented multiple imputations for missing data.

### Variables

#### Cognitive function

In line with the USA Health and Retirement Study, the items for cognitive function in CHARLS were components of the Telephone Interview of Cognitive Status (TICS) battery [31], which is a telephone version of the Mini-Mental State Examination validated for measuring one’s cognitive function [32]. Prior studies have suggested measuring cognitive function by memory and mental status [19,23]. CHARLS assessed memory by testing one’s skills in immediate and delayed word recall (ranging from 0-20 points) and mental status based on time orientation, picture drawing, and numeric ability (ranging from 0-11 points). We measured time orientation by asking respondents to name the date (year, month, and day), season, and day of the week, picture drawing by asking respondents to accurately redraw a previously shown picture, and numeric ability by having them subtract seven from 100 consecutively for five times. The total cognitive function score was obtained by summing the components and ranged from 0 to 31, with higher scores indicating better cognitive function.

#### Sensory impairment and eye diseases

We assessed visual impairments, including distance visual and near visual impairment, by asking, “Is your eyesight for seeing things at a distance excellent, very good, good, fair, or poor?” and “How good is your eyesight for seeing things up close, like reading ordinary newspaper print? Would you say your eyesight for seeing things up close is excellent, very good, good, fair, or poor?” For hearing impairment, CHARLS asked respondents “Is your hearing excellent, very good, good, fair, or poor?” We identified respondents as distance/near visual impairment if they reported poor eyesight for seeing things at a distance/up close. We classified reporting poor hearing as hearing impairment.

Eye diseases included cataracts, diagnosed if respondents reported that they had cataract surgery, and glaucoma, diagnosed if a doctor ever treated them for glaucoma.

#### Social participation

The social activities from CHARLS included: interaction with friends, playing mah-jong, chess, and cards, or going to a community club, going to sports or other kinds of club, taking part in a community-related organisation, doing voluntary or charity work, and attending an educational or training course. For social participation, we asked the respondents whether they participated in any of the above social activities, dichotomising them into no/yes.

#### Control variables

Control variables included three aspects of socio-demographic characteristics, health behaviours (smoking (no/yes) and alcohol consumption (no/yes)), health outcomes (chronic diseases (none, 1 type, 2 types, ≥3 types) and instrumental activities of daily living (IADLs)), and time variables. For socio-demographic characteristics, we included gender (male, female), age, residency (rural, urban), education (illiterate, primary school and lower, middle school, senior middle school and higher), marital status (single, partnered), retirement (no, yes), living near children (no, yes), and per capita household income (total household income/ number of people in household). We defined IADL impairment as having difficulty in one or more of five IADL items.

### Data analysis

We described the sample characteristics using means and standard deviation (SD) for continuous data and percentages for categorical data. We examined the variables and the differences in proportions using a *t*-test for continuous and a χ^2^ test for categorical variables. After adjusting for potentially confounding variables, we used a longitudinal linear fixed-effects regression model and distribution regression to explore the association of sensory impairment and eye diseases with cognitive function. On this basis, we assessed the mediating effect of social participation in their relationship.

*Cognitive function_it_* = *μ_t_* + *cX_it_* + *βδ_it_* + *ε_it_*

*Social participation_it_* = *μ_t_* + *aX_it_* + *βδ_it_* + *ε_it_*

*Cognitive function_it_* = *μ_t_* + *c’X_it_* + *bSocial participation_it_* + *βδ_it_* + *ε_it_*

where *Cognitive function_it_* indicates the cognitive performance of individual *i* at time *t* and *Social participation_it_* represents the social activities of individual *i* at time *t*. *X_it_* represents the sensory impairment and eye diseases for individual *i* at time *t*, *δ_it_* denotes time-varying variables, such as health behaviours and health outcome variables, and *μ_t_* captures year-specific effects, while *Ƹ_it_* characterizes random error.

We used the *F*-test and Hausman test for model selection among the random-effects model, ordinary least squares (OLS), and fixed-effects model; their results were statistically significant (*P* < 0.001), indicating that the fixed-effects model was a better choice. We presented the results using correlation coefficients (β) and 95% confidence intervals (95% CIs). We standardised all variables before data analysis. We performed all analyses using R, version 4.2.2.

## RESULTS

### Basic characteristics of respondents

**Table 1** shows the basic characteristics of the respondents. Amongst the 2876 respondents, their mean age was 72.73 ± 7.97 years, a greater proportion were female, living in rural areas, with illiterate education, living with a partner and near children. In addition, the prevalence of near visual impairment, distance visual impairment and hearing impairment was 25.80%, 33.28% and 27.54%, respectively. The prevalence of cataracts and glaucoma was 1.74% and 2.05%, respectively. Respondents without sensory impairment or eye diseases had higher cognition scores than those with sensory impairments or eye diseases.

**Table 1:** Sample characteristics at baseline

Variables	Total = 2876, n (%)	Cognition scores, mean (SD)	*P*
**Near visual impairment**			<0.001
Yes	742 (25.80)	11.49 (4.72)	
No	2134 (74.20)	12.71 (5.27)	
**Distance visual impairment**			<0.001
Yes	957 (33.28)	11.38 (4.94)	
No	1919 (66.72)	12.90 (5.20)	
**Cataract**			0.029
Yes	50 (1.74)	10.24 (5.32)	
No	2826 (98.26)	12.43 (5.15)	
**Glaucoma**			0.023
Yes	59 (2.05)	10.39 (5.15)	
No	2817 (97.95)	12.44 (5.16)	
**Hearing impairment**			<0.001
Yes	792 (27.54)	11.99 (4.87)	
No	2084 (72.46)	12.55 (5.26)	
**Gender**			<0.001
Male	1396 (48.54)	13.18 (5.12)	
Female	1480 (51.46)	11.66 (5.09)	
**Residency**			<0.001
Urban	1190 (41.38)	13.65 (5.38)	
Rural	1686 (58.62)	11.51 (4.80)	
**Education level**			<0.001
Illiterate	1266 (44.02)	10.13 (4.15)	
Primary school or lower	1101 (38.28)	13.07 (5.03)	
Middle school	309 (10.75)	16.11 (4.94)	
High school or higher	200 (6.95)	17.31 (4.08)	
**Martial status**			<0.001
Divorced/widowed/single	1104 (38.39)	11.46 (4.86)	
Married/partnered	1772 (61.61)	12.98 (5.26)	
**Age**	72.73 (7.97)	-	<0.001
**Per capita household income**			<0.001
Median (IQR)	5903.67 (1637.50-19800.00)	-	
**Smoking**			0.005
No	1527 (53.09)	12.07 (5.21)	
Yes	1349 (46.91)	12.77 (5.09)	
**Alcohol consumption**			0.104
No	1578 (54.87)	12.10 (5.16)	
Yes	1298 (45.13)	12.76 (5.15)	
**IADLs**			<0.001
no difficulty	1472 (51.18)	13.54 (5.30)	
some difficulty	1404 (48.82)	11.20 (4.73)	
**Living near children**			0.013
No	1176 (40.89)	12.78 (5.28)	
Yes	1700 (59.11)	12.13 (5.06)	
**Retirement**			<0.001
No	932 (32.41)	12.04 (5.11)	
Yes	1944 (67.59)	12.57 (5.18)	
**Chronic diseases**			0.149
None	217 (7.54)	12.26 (5.03)	
One type	498 (17.32)	11.87 (5.11)	
Two types	574 (19.96)	12.45 (5.22)	
Three types or more	1587 (55.18)	12.56 (5.17)	

SD – standard deviation, IQR – interquartile range, IADLs – instrumental activities of daily living

### The association between visual impairment and cognitive function and the mediating role of social participation

Near and distance visual impairment were strongly associated with a poor memory and mental status. Compared with near visual impairment, distance visual impairment was associated with a 1.7 times higher likelihood of cognitive decline (β = -0.051; 95% CI = -0.065, -0.036) (**Table 2**).

**Table 2:** Associations of sensory impairments and eye diseases with cognitive function in elderly Chinese*

	Model 1, β (95% CI)			Model 2, β (95% CI)	Model 3, β (95% CI)		
	**Cognition**	**Memory**	**Mental status**	**Social participation**	**Cognition**	**Memory**	**Mental status**
**Near visual impairment (ref: no)**							
Yes	-0.030 (-0.044, -0.016)§	-0.024 (-0.039, -0.009)‡	-0.023 (-0.038, -0.007)‡	-0.042 (-0.058, -0.026)§	-0.027 (-0.041, -0.013)§	-0.021 (-0.035, -0.006)‡	-0.022 (-0.037, -0.006)‡
Social participation	-	-	-	-	0.071 (0.056, 0.085)§	0.075 (0.060, 0.090)§	0.029 (0.013, 0.044)§
**Distance visual impairment (ref: no)**							
Yes	-0.051 (-0.065, -0.036)§	-0.056 (-0.071, -0.040)§	-0.019 (-0.034, -0.003)†	-0.043 (-0.063, -0.028)§	-0.048 (-0.062, -0.033)§	-0.052 (-0.068, -0.037)§	-0.017 (-0.033, -0.002)†
Social participation	-	-	-	-	0.070 (0.055, 0.084)§	0.074 (0.059, 0.089)§	0.029 (0.013, 0.044)§
**Cataracts (ref: no)**							
Yes	-0.039 (-0.053, -0.025)§	-0.030 (-0.045, -0.016)§	-0.032 (-0.047, -0.017)§	0.025 (0.009, 0.040)‡	-0.041 (-0.055, -0.027)§	-0.032 (-0.047, -0.018)§	-0.033 (-0.048, -0.017)§
Social participation	-	-	-	-	0.073 (0.058, 0.087)§	0.077 (0.062, 0.092)§	0.030 (0.015, 0.046)§
**Glaucoma (ref: no)**							
Yes	-0.021 (-0.035, -0.007)‡	-0.020 (-0.035, -0.006)‡	-0.011 (-0.027, 0.003)	-0.002 (-0.018, 0.012)	-0.021 (-0.035, -0.007)‡	-0.020 (-0.035, -0.006)‡	-0.011 (-0.027, 0.003)
Social participation	-	-	-	-	0.072 (0.057, 0.086)§	0.076 (0.061, 0.091)§	0.030 (0.014, 0.045)§
**Hearing impairment (ref: no)**							
Yes	-0.030 (-0.045, -0.016)§	-0.046 (-0.061, -0.031)§	0.005 (-0.010, 0.020)	-0.027 (-0.043, -0.010)‡	-0.028 (-0.043, -0.014)§	-0.044 (-0.059, -0.029)§	0.006 (-0.009, 0.021)
Social participation	-	-	-	-	0.071 (0.057, 0.085)§	0.075 (0.060, 0.090)§	0.030 (0.014, 0.045)§

CI – confidence interval

*Model 1: *Cognitive function_it_* = *μ_t_*+*cX_it_*+*βδ_it_*+*ε_it_*, Model 2: *Social participation_it_* = *μ_t_*+*aX_it_*+*βδ_it_*+*ε_it_*, Model 3: *Cognitive function_it_* = *μ_t_*+*c’X_it_*+*bSocial participation_it_* +*βδ_it_*+*ε_it_*. Model 1 and Model 2 were adjusted for age, education level, marital status, retirement, smoking, alcohol consumption, living near children, instrumental activities of daily living (IADLs), average income, and chronic diseases. Model 3 was adjusted for social participation, and variables in Model 1 and Model 2.

†*P* < 0.05.

‡*P* < 0.01.

§*P* < 0.001.

We found a significant indirect effect between visual impairment and cognitive function. Additionally, the direct effects of near and distance visual impairment on cognitive function were -0.027 (95% CI = -0.041, -0.013) and -0.048 (95% CI = -0.062, -0.033), respectively (**Table 3**). Social participation partially mediated the relationship between visual impairments and cognitive function in elderly Chinese. Moreover, the indirect effect of social participation on near visual impairment and cognitive function accounted for 9.94% of the total effect, while its indirect effect on distance visual impairment and cognitive function accounted for 5.90% of the total effect.

**Table 3:** Coefficients of mediation analysis

Pathway	Estimate	95% CI	*P*-value
**Sensory impairments**			
X1 to M to Y			
*Total effect*	-0.030	-0.044, -0.016	<0.001
*Direct effect*	-0.027	-0.041, -0.013	<0.001
*Indirect effect – X1 to M*	-0.042	-0.058, -0.026	<0.001
*Indirect effect – M to Y*	0.071	0.056, 0.085	<0.001
X2 to M to Y			
*Total effect*	-0.051	-0.065, -0.036	<0.001
*Direct effect*	-0.048	-0.062, -0.033	<0.001
*Indirect effect – X2 to M*	-0.043	-0.063, -0.028	<0.001
*Indirect effect – M to Y*	0.070	0.055, 0.084	<0.001
X3 to M to Y			
*Total effect*	-0.030	-0.045, -0.016	<0.001
*Direct effect*	-0.028	-0.043, -0.014	<0.001
*Indirect effect – X3 to M*	-0.027	-0.043, -0.010	<0.01
*Indirect effect – M to Y*	0.071	0.057, 0.085	<0.001
**Eye diseases**			
X4 to M to Y			
*Total effect*	-0.039	-0.053, -0.025	<0.001
*Direct effect*	-0.041	-0.055, -0.027	<0.001
*Indirect effect – X4 to M*	0.025	0.009, 0.040	<0.01
*Indirect effect – M to Y*	0.073	0.058, 0.087	<0.001
X5 to M to Y			
*Total effect*	-0.021	-0.035, -0.007	<0.01
*Direct effect*	-0.021	-0.035, -0.007	<0.01
*Indirect effect – X5 to M*	-0.002	-0.018, 0.012	0.717
*Indirect effect – M to Y*	0.072	0.057, 0.086	<0.001

CI – confidence interval, X1 – near visual impairment, X2 – distance visual impairment, X3 – hearing impairment, X4 – cataracts, X5 – glaucoma, M – social participation, Y – cognitive function

### The association between hearing impairment and cognitive function and the mediating role of social participation

We found hearing impairment to be significantly associated with bad memory (β = -0.046; 95% CI = -0.065, -0.036) but not mental status (β = 0.005; 95% CI = -0.010, 0.020) (**Table 2**). The indirect effect of hearing impairment on cognitive function was statistically significant (**Table 3**), while its direct effect on cognitive function was -0.028 (95% CI = -0.043, -0.014). Social participation partially mediated the relationship between hearing impairment and cognitive function in elderly Chinese. Moreover, the indirect effect of social participation on hearing impairment and cognitive function accounted for 6.39% of the total effect.

### The association between eye diseases and cognitive function and the mediating role of social participation

Cataracts were strongly associated with poor memory (β = -0.030; 95% CI = -0.045, -0.016) and mental status (β = -0.032; 95% CI = -0.047, -0.017), while glaucoma was significantly associated with the former (β = -0.020; 95% CI = -0.035, -0.006) but not the latter (β = -0.011; 95% CI = -0.027, 0.003) (**Table 2**).

We found a significant indirect effect on cognitive function for cataracts, but not for glaucoma. The direct effect of cataracts on cognitive function was -0.041 (95% CI = -0.055, -0.027), and that of glaucoma on cognitive function was -0.021 (95% CI = -0.035, -0.007). Social participation partially mediated the relationship between cataracts and cognitive function in elderly Chinese. Additionally, the indirect effect of social participation on cataracts and cognitive function accounted for 4.68% of the total effect (**Table 3**).

### Compare the influences of sensory impairments and eye diseases on cognitive function

Compared with individuals who suffered from eye diseases, those with sensory impairments took part in fewer social activities (**Table 3**). Simultaneously, social participation contributed to a total indirect effect of 22.23% on the relationship between sensory impairments and cognitive function in elderly Chinese. Compared with those who suffered from eye diseases, those who suffered from sensory impairments reported a 4.8 times higher likelihood of cognitive decline. Additionally, those who suffered from distance visual impairment were affected by limited social participation and reported faster cognitive decline than those who suffered from near visual and hearing impairment. Meanwhile, we found a significant negative correlation between cataracts and social participation. Furthermore, compared with those who suffered from glaucoma, those who suffered from cataracts reported a 1.9 times higher likelihood of cognitive decline.

Overall, hypotheses 1a, 1b, 2a, 3, and 4a were supported, 4b was partially supported, while 2b was unsupported in our study (**Table 4**).

**Table 4:** Results of hypotheses testing

Hypothesis	Results
H1a: Sensory impairments to cognitive function (negative)	Supported
H1b: Eye diseases to cognitive function (negative)	Supported
H2a: Sensory impairments to social participation (negative)	Supported
H2b: Eye diseases to social participation (negative)	Unsupported
H3: Social participation to cognitive function (positive)	Supported
H4a: Mediating effect of social participation on the relationship between sensory impairments and cognitive function	Supported
H4b: Mediating effect of social participation on the relationship between eye diseases and cognitive function	Partially supported

H – hypothesis

## DISCUSSION

We used three waves of a longitudinal survey to examine the impact of sensory impairments and eye diseases on cognitive function in elderly Chinese. To further explore the underlying mechanism, we introduced social participation to investigate its mediating effects in the relationships between sensory impairments, eye diseases, and cognitive function among Chinese older people. We found that visual impairment and cataracts were associated with poor cognitive function in both memory and mental status, while hearing impairment and glaucoma were only associated with a bad memory. We also found that visual impairment, hearing impairment, and cataracts influence cognitive function partly via social participation.

Our findings support hypotheses 1a and 1b that Chinese older people who suffered from sensory impairments or eye diseases developed worse cognitive function over time. We found that distance and near visual impairment were associated with poor cognitive function in both memory and mental status, whereas hearing impairment was only associated with a bad memory. Our findings are consistent with the information degradation hypothesis in that decreased perceptual information can decrease neuronal input, potentially accelerating neurodegeneration, and resulting in cognitive decline among older adults [15]. The common cause hypothesis indicates that sensory impairments and cognitive decline may be caused by a common pathological process with the degeneration of the nervous system. The cognitive load on perception hypothesis states that individuals under high cognitive load will suffer from sensory impairments due to limited cognitive resources. Further studies are needed as we cannot test the common cause and the cognitive load on perception hypotheses. However, hearing impairment may result in long-term auditory deprivation or degraded auditory input and increased compensatory effort to remember sequences of spoken digits, which are detrimental to one’s brain function regarding digit and verbal memory [33]. This compensatory effort may use up limited cognitive resources, resulting in an apparent decrement in memory [34]. Furthermore, individuals with visual impairment or hearing impairment are more likely to be associated with anxiety and depression, leading to anhedonia and poor sleep, which may impair one’s cognitive function [35].

Similarly, we also found individuals with cataracts suffered from poor cognitive function in both memory and mental status, but individuals with glaucoma only suffered from bad memory. Previous studies have shown that the incidence of cataracts increases with age, reaching 70.5% in people over 75 years old [36,37]. Cataract-related visual impairment may decrease neuronal input, potentially accelerating neurodegeneration or magnifying the effect of neurodegeneration through cortical atrophy [15], all linked to cognitive decline. Besides, elderly people with cataracts may avoid social contact, exercise, and activity due to blurred vision, which may result in a deterioration of physiological functions and the parts of the brain associated with memory and mental processes [38]. However, it has been reported that glaucoma is a memory-related neurodegenerative disease that affects the central regions of the visual system due to trans-synaptic degeneration in individuals with glaucoma [39]. Furthermore, cataracts can be treated by surgery, but not glaucoma. Cataract surgery helps to recover the vision of cataract individuals, which could contribute to enhancing social participation. Thus, hypothesis 2b was unsupported in this study. Simultaneously, the increased quantity and quality of light in social engagement may be associated with a lower risk of cognitive impairment [40]. Intrinsically photosensitive retinal ganglion cells (ipRGCs) project to multiple areas of the brain, and their excitation may trigger widespread cortical activity, which has been shown to be associated with better cognitive function [41].

Simultaneously, we found that social participation partially mediated the relationships between visual impairment, hearing impairment, cataract, and cognitive function, but not the relationship between glaucoma and cognitive function. Our findings support hypotheses 2a, 3, 4a and, in part, 4b, and are in line with previous research [42,43]. Although the pathophysiology of the links between sensory impairments, eye diseases, and cognitive function are complex, research suggests that visually impaired individuals have more difficulties in performing daily activities, such as reading and seeing objects. According to the role theory [22], the role loss perspective posits that the sudden role loss individuals encounter when they move into older age may result in negative effects on well-being and quality of life among older adults. Moreover, individuals with visual impairment or hearing impairment may be more likely to have limited social participation and interactions with others [24]. The cognitive reserve hypothesis suggests that reduced social engagement in cognitively stimulating activities may lead to brain reserve deficits [44], and impair the function of parts of the brain related to temporal orientation, visuospatial [45], and mathematical performance [46], resulting in poor memory and mental status.

Interestingly, we found that individuals with sensory impairments participated in fewer social activities and had poorer cognitive function than those with eye diseases. This might be explained by the increase in the risk of visual and hearing with age; visual and hearing impairments are chronic conditions that are not conducive to participating in social activities, and they increase the risk of cognitive decline [8]. However, clinical evidence shows that eye surgery resulting in improved vision is associated with active social participation and improved cognitive function [8]. Thus, sensory impairments were associated with a higher likelihood of cognitive decline than eye diseases. We also found a more significant influence of distance visual impairment on cognitive function than near visual and hearing impairment. This may be related to the prevalence of distance visual impairment in the elderly, which is consistent with our results [15,47]. Previous research pointed out that distance visual impairment was gradual and the prevalence increased with age [48]. The World Health Organization estimates that about 189 million people are currently living with distance visual impairment [49], with most of those affected residing in developing countries [50]. Moreover, a population study based on communities of older adults in the USA indicated that 20% of the elderly experience significant distance visual impairment [47]. The differences in the influences of distance visual impairment, near visual impairment, hearing impairment, and cataracts on cognitive function have implications for policymakers, helping them use proper measures and therapy appropriately for addressing key issues.

This study has several strengths. It fills a gap in literature on biomedical-psychological-social linkage by focusing on cognitive function in a developing country. Second, we used a national representative sample, making our findings more generalisable. Third, the use of longitudinal linear fixed-effects regression model and distribution regression allowed us to establish the causal relationships between sensory impairments, eye diseases, and cognitive function. Fourth, we investigated the mediating effect of social participation in the relationships between sensory impairments, eye diseases and cognitive decline, further highlighting the importance of social participation.

However, some limitations should also be considered. First, the data on sensory impairments and eye diseases were self-reported, without any comparative objective data, which may result in recall bias due to hypomnesia in one’s older age. Second, we did not investigate whether assistive devices (e.g. hearing aids, glasses) could potentially mitigate cognitive function. Third, the study participants may not have fully represented the elderly people in China, as the CHARLS team conducted the survey among community-dwelling residents, and those who live in a nursing home were not included. Future studies should attempt to better understand the impacts of sensory impairments and eye diseases on cognitive function in China’s context by applying a more sophisticated design.

## CONCLUSIONS

Based on representative surveys, we observed visual impairment and cataracts were associated with poor cognitive function in both memory and mental status, whereas hearing impairment and glaucoma were only associated with a bad memory. After incorporating social participation as a mediating variable, we determined a mechanism underlying the effects of social participation in the relationship between visual impairment, hearing impairment, cataracts, and cognitive function in elderly Chinese. We recommend early interventions for sensory impairments and eye diseases that may improve the cognitive function of elderly people.
